# F178. NEUROANATOMICAL PROFILES OF TREATMENT-RESISTANCE IN PATIENTS WITH SCHIZOPHRENIA

**DOI:** 10.1093/schbul/sby017.709

**Published:** 2018-04-01

**Authors:** Eric Plitman, Yusuke Iwata, Shinichiro Nakajima, Jun Ku Chung, Raihaan Patel, Fernando Caravaggio, Julia Kim, Vincenzo De Luca, Sofia Chavez, Gary Remington, M Mallar Chakravarty, Philip Gerretsen, Ariel Graff-Guerrero

**Affiliations:** 1 Centre for Addiction and Mental Health, University of Toronto; 2 Douglas Mental Health University Institute, McGill University

## Abstract

**Background:**

About 20 to 35% of patients with schizophrenia show partial or no response to standard first-line antipsychotic treatment and are thus believed to have treatment-resistant schizophrenia (TRS). Notably, the antipsychotic clozapine (CLZ) has been reported to have superior efficacy compared to other agents for the treatment of TRS. However, a subset of patients still do not respond to CLZ treatment and are thus considered to have ultra-treatment-resistant schizophrenia (UTRS). Overall, the pathophysiology associated with UTRS appears to be different than TRS, yet both remain elusive. In light of the unknown factors underlying UTRS and TRS, along with the widely reported structural alterations that exist in patients with schizophrenia, our study aimed to examine subcortical structure volumes and cortical thickness in patients with UTRS, patients with TRS responding to CLZ (henceforth, TRS), patients responding to a first-line antipsychotic (treatment non-resistant schizophrenia (TnRS)), and healthy controls (HC). We hypothesized that deficits in subcortical structure volumes and cortical thickness would exist within the UTRS group compared to other groups.

**Methods:**

As of December 2017, the sample consisted of a total of 94 participants, including 24 patients with UTRS, 24 patients with TRS, 21 patients with TnRS, and 25 HCs. Participants underwent a 3-dimensional T1-weighted scan in a 3T MRI machine. The MAGeT-Brain segmentation algorithm was used to acquire volumes of the thalamus, striatum, globus pallidus, hippocampus, and amygdala. Cortical thickness was estimated using the CIVET processing pipeline. Total brain volume was obtained using the BEaST method. Group comparisons were performed using analyses of covariance and post-hoc comparisons.

**Results:**

Group volumetric differences were identified bilaterally within the thalamus, striatum, and globus pallidus (p<0.01). Post-hoc investigations revealed that bilateral thalamic volumes were smaller in the UTRS group compared to the HC group (p<0.01), bilateral striatal volumes were larger in the TnRS group compared to the UTRS and HC groups (p<0.01), and bilateral globus pallidus volumes were larger in the TnRS group compared to the HC group (p<0.01). No differences in hippocampal, amygdala, or total brain volume were observed. At a 5% false discovery rate, widespread cortical thinning was identified in both the UTRS and TRS groups compared to the TnRS and HC groups; this effect was stronger and more diffuse in the UTRS group.

**Discussion:**

Our findings suggest that thalamic volume deficits might be a distinct feature of UTRS. Contrastingly, striatal and globus pallidus volume enlargement may be associated with first-line antipsychotic response or treatment. Cortical thinning is apparent in both the UTRS and TRS groups. In many cases, structural compromise appears to follow a continuum of response, whereby deficits are most severe in UTRS patients, followed by TRS patients, who are followed by TnRS patients and HCs.

